# Carbon footprints of greenhouse gas mitigation measures for a grass-based beef cattle finishing system in the UK

**DOI:** 10.1007/s11367-025-02428-9

**Published:** 2025-01-14

**Authors:** Asma Jebari, Taro Takahashi, Michael R. F. Lee, Adrian L. Collins, Kevin Coleman, Alison Carswell, Carmen Segura, Laura Cardenas, Graham A. McAuliffe

**Affiliations:** 1 Net Zero and Resilient Farming, Rothamsted Research, North Wyke, Okehampton, Devon EX20 2SB UK; 2 Agri-Food and Biosciences Institute, Hillsborough, BT26 6DR UK; 3 University of Bristol, Langford, BS40 5DU UK; 4 School of Sustainable Food and Farming, Harper Adams University, Edgmond, Shropshire TF10 8NB UK; 5 Net Zero and Resilient Farming, Rothamsted Research, Harpenden, AL5 2JQ UK; 6 Harper Food Innovation, Harper Adams University, Edgmond, Shropshire TF10 8NB UK

**Keywords:** Soil organic carbon, Carbon footprint, Extensification, Anaerobic digestion, Nitrification inhibitor

## Abstract

**Purpose:**

Agri-food systems across the globe are faced with the challenge of reducing their supply-chain emissions of greenhouse gases (GHGs) such as nitrous oxide (N_2_O), carbon dioxide (CO_2_), and methane (CH_4_). For instance, 10% of the UK’s GHG emissions are generated by agriculture, and ~ 56% of these are generated by livestock production. Numerous mitigation measures are being proposed to reduce GHG emissions from ruminants (representing 70 to 80% of total livestock emissions), particularly from beef cattle (presenting 30–40% of total livestock emissions).

**Methods:**

To explore such potential, first, a business-as-usual (BAU) partial cradle-to-finishing farmgate scale modelling framework was developed. The BAU systems (i.e. steady-state productivity based on primary data from the North Wyke Farm Platform) were built using ensemble modelling wherein the RothC process-based soil organic carbon (SOC) model was integrated into the life cycle assessment (LCA) framework to conduct a trade-off analysis related to mitigation measures applicable to the study system. Potential mitigation measures were applied to the BAU scenario. The interventions assessed included: (i) extensification; (ii) adopting anaerobic digestion technology; and (iii) the use of the nitrification inhibitor DCD and substitution of fertiliser nitrogen with symbiotically fixed nitrogen from legumes.

**Results:**

The partial carbon footprint for 1 kg of beef liveweight gain leaving the farmgate *could* be reduced by 7.5%, 12%, or 26% by adopting nitrification inhibitors, white clover introduction (pending establishment success), and anaerobic digestion for manure management, respectively.

**Conclusions:**

The findings highlight the importance of including emissions beyond the farmgate level to analyse the carbon footprint of different management scenarios in order to assess the sustainability of agri-food production systems.

**Supplementary Information:**

The online version contains supplementary material available at 10.1007/s11367-025-02428-9.

## Introduction

In 2018, the largest share of global greenhouse gas (GHG) emissions was reported to originate from the energy sector (34%), followed by industry (24%), Agriculture, Forestry and Other Land Use (21%), and transport 14% (Lamb et al. 2021). The global demand for beef has been rapidly increasing (Research and markets 2023), raising concerns about concomitant climate change impacts (Clark et al. 2020; Leip et al. 2015; Springmann et al. 2018). Globally, beef and dairy production contributes over 70% of livestock GHG emissions, which collectively contribute to ~ 6.3 Gt CO_2_-eq/year (Gerber et al. 2013). As a consequence, domesticated bovine production, particularly beef and dairy, has become a focal point in terms of mitigating the GHGs produced by the livestock sector, notably enteric methane produced by ruminants (Beauchemin et al. 2020). The utility of beef GHG mitigation strategies and increased production efficiency is not without their concerns (Garnett et al. 2017). Such reservations can be attributed, in part, to rumen microbial fermentation which, although enabling the ruminant to utilise otherwise undigestible fibre-rich feeds as a by-product, also forms methane (CH_4_).

In the UK, despite robust evidence and method development surrounding the technically feasible impacts of agricultural mitigation measures using marginal abatement cost curves (MACC; Eory et al. 2018), few studies have progressed assessing mitigation measures for reducing the climate change impacts of grassland-based beef production using LCA. For instance, the impact of transitioning from permanent pasture to novel swards on nitrogen use efficiency (NUE) was assessed using nitrogen and carbon budgets of beef and sheep production in England (Carswell et al. 2019). Using Scotland as a case study, Kamilaris et al. (2020) combined a bio-economic simulation model and farm-level carbon footprinting tool to study the environmental impact of a range of beef production scenarios and trade-offs generated between mitigating emissions and increasing farm profitability. The authors highlighted that medium duration (i.e. 18–24 months) grassland-based beef production systems in Scotland were found to achieve a balance between financial returns and environmental performance. A study comprising sites in different nations in the UK (Cardenas et al. 2019) found that NUE depended on the type of N fertiliser, and although NUE increased with fertiliser rate, so did the emissions of N_2_O. Measures such as application of inhibitors and splitting N application were effective in improving NUE in these grasslands.

More recently, using qualitative methods, different mitigation measures in the UK agricultural sector were synthesised based on the existing scientific literature and elicitation of expert opinions (Buckingham et al. 2023; Jebari et al. 2023). Nevertheless, due to system interactions, mitigation practices that reduce emissions in one stage may increase emissions elsewhere, and mitigation practices must therefore be evaluated at the whole farm level (Montes et al. 2013). To the best of our knowledge, there have not been studies in the UK investigating multiple mitigation measures via a data-driven scenario analysis with respect to grassland, grazing, and manure management of beef production systems using LCA. The sophistication of LCA has evolved rapidly in recent years (e.g. McLaren et al. 2021) and arguably has become the de facto gold standard means to quantify environmental footprints of agri-food commodity supply chains in silico over the last few decades (Igos et al. 2019). Indeed, one such example of advancing LCA is how high-resolution data can catapult the method through the development of a novel approach to life cycle inventory analysis (LCI) by calculating GHG emissions of individual finishing cattle thus enabling the assessment of dependent (emissions) and independent variables (e.g. growth rates, breeds, and sex) to identify the most promising areas in terms of livestock management and GHG mitigation. Here, McAuliffe et al. (2018) found that average daily weight gains (ADG) were strongly (and negatively) correlated variables to individual animal carbon footprints, and that using averaged livestock performance data may underestimate emission intensities due to insufficient consideration being given to poorly performing animals, whose emissions become exponentially greater as their ADG decreases.

Moreover, despite the lack of reported impacts concerning soil organic carbon (SOC) changes in LCA of agricultural products (Jebari et al. 2022), SOC accumulation is expected to hold a major potential to mitigate agricultural GHG emissions (Petersen et al. 2013). To improve impact assessment estimates, LCA studies should therefore uniformly include SOC changes and be conducted over longer timespans to assess the stability of added C storage for a full accounting of agricultural GHG emissions (e.g. including beef production) (Cusack et al. 2021).

Goglio et al. (2015) reviewed different methods to account for SOC in agricultural LCA. In their ranking of preferences of SOC estimation methods, models were preferred to measurements. Due to inherently high spatial variability of SOC stocks and the high cost of measurements, there is little measured data available from the case studies to allow for the assessment of changes in SOC (Heikkinen et al. 2021). Conversely, different modelling approaches are already widely applied to assess changes in SOC in agriculture and have been shown to generate accurate results (e.g., Riggers et al. 2019; Jebari et al. 2021). It is worth pointing out that both a combination of direct measurements (for validation purposes) and modelling (at larger scales) can greatly help define the efficacy of different land management practices in enhancing soil C sequestration (Smith et al. 2020).

Additionally, there is now a consensus that LCA studies must acknowledge uncertainties inherent within production systems to ensure their scientific robustness (Igos et al. 2019). Theoretically speaking, uncertainties in carbon footprint estimates can arise from either of the two computational stages of LCA, namely, life cycle inventory analysis (LCI) and life cycle impact assessment (LCIA). Within the context of livestock production systems, however, the choice of LCIA methodology has been shown to impart negligible effects on global warming potential as long as the timescale of analysis (e.g. 20 years, 100 years, or 500 years) is clearly defined (Reckmann 2013). Uncertainties associated with LCI, on the other hand, have considerable impacts on environmental footprints arising from different farming systems (McAuliffe et al. 2017) and are also of relevance to a large population of practitioners around the world. Nonetheless, LCA studies often omit rigorous evaluation of system-level uncertainties (Imbeault-Tétreault et al. 2013).

Given the background described above, this study aimed to estimate the partial LCA for a beef production system under humid temperate conditions in southwest England, by derivation of total emissions for each individual animal from cradle to gate. More specifically, the main objectives were to identify the major hotspots relevant for GHG emissions and assess possible mitigation alternatives. To do so, SOC changes were included in the LCI, as well as an uncertainty analysis derived from the different mitigation measures included in the scenarios.

## Methods

The results presented herein were produced following the fundamental LCA theories and recommendations provided by ISO 14040 (2006) and ISO 14044 (2006) guidelines in a ‘cradle to gate’ approach. We focus exclusively on the finishing stage, as described in Section 2.1. Mitigation measures downstream from the farmgate were not considered, as the majority of GHG emissions produced in ruminant systems are typically emitted during the primary production stage (Asem-Hiablie et al. 2019; Verge et al. 2013; Seo et al. 2017). The analysis was limited to the global warming. Henceforth, any references to ‘global warming’ or ‘footprints’ are GHG-centric solely (i.e. we only consider the impact category ‘GWP100’). Socioeconomic aspects and trade-offs such as capital investment, maintenance costs, and willingness to adopt various mitigation measures by farmers were considered in a separate study (Jebari et al. 2024).

### Goal and scope

The aim of the study was to assess the efficacy of various mitigation measures on the climate change impact of a grassland-based beef production system in Southwest England. For each mitigation measure derived from Jebari et al. (2023), a separate LCI was conducted to ensure that all effects of introducing novel management interventions or technologies were captured as accurately as possible. The attributional LCA modelling theory was followed as opposed to consequential theory, which considers changes in supply and demand for a product or service.

### System boundaries and functional unit

**Fig. 1:**
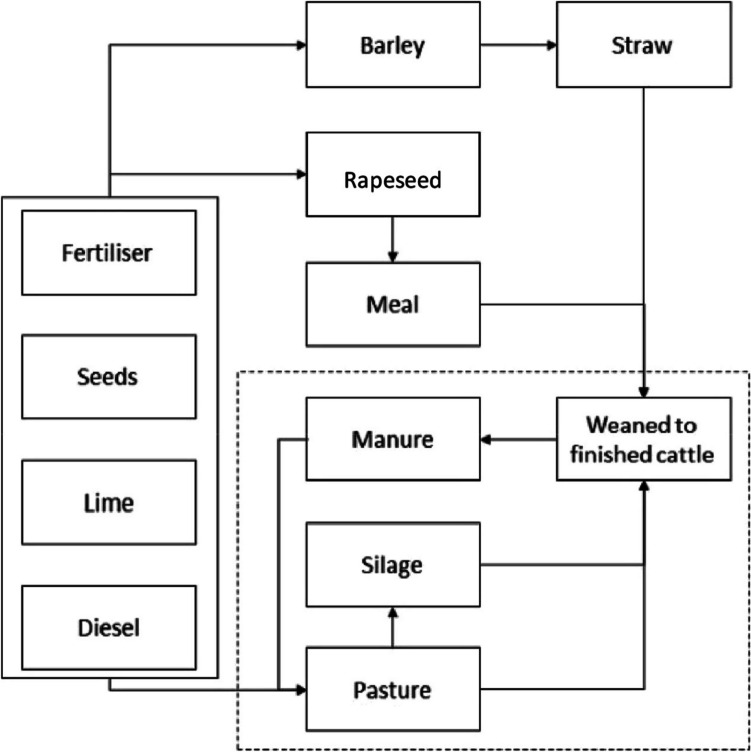
Study system boundary adjusted from previous work to reflect the feed inputs within the temporal boundary (i.e. 2016 grazing cattle born in 2015). The dashed line represents the North Wyke Farm Platform (NWFP) which is the foreground process under direct examination using primary data (see acknowledgements for access to underlying open access data used to produce the BAU inventory) (McAuliffe et al. 2018)

The system boundary was defined as ‘a partial cradle to finishing farmgate’ for the beef system maintained on the permanent pasture ‘farmlet’ of the North Wyke Farm Platform (NWFP; Fig. 1). ‘Partial’ is specified here since the suckler herd is outside of the system boundary, indicated by the liveweight gain functional unit compared to a ‘full’ cradle-to-gate analysis whereby the functional unit would most likely be liveweight, i.e. from birth to finishing, rather than from ‘weaned to finishing’. The suckler system was excluded from our study, as the cow-calf operation is not part of the permanent pasture of the NWFP, and the field data recording is at lower resolution. As a functional unit, the production of 1 kg of live weight gain (LWG) (between entering and leaving the finishing operation) was adopted at the farmgate, to allow comparability with other studies. The direct or primary emissions were those generated within the farm system (on-farm), and the secondary off-farm emissions are those upstream emissions related to the production and transport of imported resources such as feed, fertiliser, and soil amendments. The system boundary was therefore set to cover all operations from production of raw materials to departure of ready-to-slaughter animals from the finishing enterprise (Fig. 1).

### Description of the system

The study was carried out on the NWFP, located in Devon, southwest England (50°46′10″N, 3°54′05″W). The NWFP consists of three different cattle-sheep/animal farming systems. These are the following: (1) a permanent pasture; (2) a mix with legumes/clover; and (3) a high sugar and deep-rooted grass system. In this study, the permanent pasture (known as ‘the green farmlet’; 21 ha) was selected as the baseline scenario. Information on the NWFP design concept and operation is provided in Orr et al. (2016), Takahashi et al. (2018), and Hawkins et al. (2023). The soil is a clay loam topsoil (with 36% clay) (Reay et al. 2022). The permanent pasture is dominated by perennial ryegrass and remained unaltered (Table 1). On crop establishment, the permanent pasture received standard N, P, and K fertiliser application rates, according to RB209 recommendations (AHDB 2023). The amount of fertilisers used is detailed in Table 1.

**Table 1:** Inventory of material inputs for the system

Parameter	Unit	Permanent pasture
Farm area	ha	21.61
Fertiliser area^a^	ha	21.24
Yield^b^	kg dry matter/ha	11,867
Fertiliser		
N	kg	3354
P	kg	257
K	kg	1198
Lime	kg	3831
Supplementary feed/bedding		
Rapeseed expeller	kg	1927
Straw^c^	kg	38,728
Transport		
Rapeseed (road)	tkm	44
Straw (road)	tkm	2424
Fertiliser (road)	tkm	3698
Pasture quality^d^		
DE	%	74.86
CP	%	23.10
Silage quality^d^		
DE	%	69.40
CP	%	15.46

^a^In the UK, there are enforced buffer zones where synthetic or organic fertilisers cannot be applied to avoid or reduce runoff and leaching of macronutrients essential for soil resilience, thereby explaining the different areas for ‘fertiliser’ (i.e. inorganic) and ‘FYM’ (farmyard manure) application

^b^Yields are calculated based on the average of multiple ‘sward’ measurements across fields comprising the permanent pasture system using a tractor equipped with a near-infrared scanner which provides the operator with real-time data on various biomass properties including yield and moisture content

^c^Used for deep bedding and farmyard manure production subsequently used as an organic fertiliser

^d^Samples were collected at the same time from fields where animals were grazing in the context of pasture and at the point of in-barn consumption in the context of silage, and subsequently analysed for fibrous fractions to calculate digestibility, and nitrogen to calculate crude protein content using wet chemistry (see McAuliffe et al. 2018 for further information)

Every autumn, 30 Charolais x Hereford-Friesian calves (average weight of 332 kg) enter the farmlet at the point of weaning. Animals are typically housed from October to April to avoid soil damage during the wet season, then moved and kept outdoors on their pasture until they reach target weights of ca. 555 kg for heifers and 620 kg for steers. Sheep also occupy the grassland as part of a rotational grazing system, although they do not share the same pasture with cattle at any given time (Orr et al. 2016). While confined, animals are fed silage comprising grasses harvested from their grazing system (i.e. permanent pasture). Depending on the quantity and quality of silage produced in any particular year, strategic supplementary feed to balance energy and protein demands may be used and recorded. The amount of pasture yield, fertilisers used, the supplementary feed, and their transport energy is illustrated in Table 1. Cattle are housed in barns deep bedded with barley (*Hordeum vulgare*) straw, and farmyard manure (FYM) produced is stored temporarily in middens (a heap or pile of FYM stored in a sheltered concrete barn) (ca. 6 months).

### Life cycle inventory

Emissions arising from livestock and pasture were estimated using the Tier 2 IPCC refined guidelines (IPCC 2019) and national emission factors sourced from the national UK GHG emissions report (Brown et al. 2018). Economic allocation was applied to the climate change impacts of both cattle and sheep and was only applied to land-based emissions, as land (and associated inputs/pollutants) was the only shared resource between both enterprises (i.e. beef and sheep). Considering that sheep manure also contributes to pasture growth (and thus indirectly facilitates cattle LWG) and vice versa (cattle manure facilitates sheep LWG), the entire environmental burdens originating from pastures were first split between the two enterprises based on economic values of products leaving the system boundary. The land shared emissions between both sheep and cattle systems were then allocated economically as follows: emissions assigned to cattle were 74%, with 26% assigned to sheep. The mass allocation was tested and showed the same results as the economic allocation, which was aligned with an earlier same-site study by McAuliffe et al. (2018).

In order to examine both temporal differences of emissions and the effects of animal heterogeneity, livestock emissions were calculated for each animal for each time period (i.e. between two weighing events) using the weighing records and digestible energy and crude protein values obtained in the methods described below. The individual emissions were summed to obtain total livestock emissions.

Inventory analysis utilised the NWFP’s high-resolution records between the weaning of the first calf and the finishing of the last calf in 2016. Among key variables, cattle LW and pasture/silage quality (digestible energy and crude protein) were both measured every 2 to 4 weeks to estimate on-farm emissions during the corresponding period (Table 1). Detailed records of all farm inputs were maintained throughout the season. These included, for example, the type and amount of fertilisers and pesticides used, the areas these products were applied to, and the minimal supplementary feeds used during housing. Table 1 provides a detailed breakdown of inputs applied to the NWFP during the temporal boundary of the study. The global warming potential associated with all stages of the feed’s life cycle were assessed from raw material through processing and transportation. The global warming included emissions from crop production, fertilisers, soil management, and energy use during feed processing and transportation. Emissions associated with background processes, such as production and transport of straw for bedding and small quantities of supplementary feeds and rapeseed (*Brassica napus*) expeller meal in 2016, were sourced from the *Agri-footprint* (V6) database (Table S1). The production of fertiliser was derived from the *World Food Life Cycle Database (WFLDB)*, and the production of seeds was derived from *ecoinvent V3* (Wernet et al. 2016).

Grasslands in the southwest of England are typically located on hilly land with soils that become waterlogged during the soil drainage season. As these lands are unsuitable for arable crop production, emissions owing to land use and land use change were not included in the present model. It is worth mentioning here that electricity use was excluded from our system boundary as previous same-site LCA work (i.e. McAuliffe et al. 2018) demonstrated that it accounted for < 1% of total GHG emissions for the permanent pasture system; this was due to the animals being outside for approximately half the year and lighting being the only notable source of energy requirements during housing months, meaning that consumption was not enough to stand out in a contribution (or hotspot) analysis. The only exception to this cut-off rule for energy consumption across scenarios (described in detail in Section 2.5) occurred during on-site anaerobic digestion of farmyard manure where energy use was indeed included; however, it remained lower than 1% of system-wide contributions, and therefore, energy remained part of ‘other’ emissions even when anaerobic digestion was considered. The carbon footprint in this study referred to the ‘partial cradle-to-gate carbon footprint’. It was calculated according to the IPCC (2021) 100-year average impact assessment method on SimaPro V8.0 (www.pre-sustainability.com). This method was based on the recent IPCC assessment report AR6 of 2021. Under this method, global warming potential (GWP) of fossil and biogenic CH_4_ in CO_2_-eq were 29.8 and 27.2, respectively, and the GWP for N_2_O was 273. The breakdown of the carbon footprints related to the different processes is illustrated in the Table S1.

#### Inclusion of SOC changes

Calculating an accurate C footprint for a management strategy is the main goal of LCAs. Since soil C sequestration is one of the primary ways to offset cattle GHG emissions, LCAs should explicitly include soil C fluxes (Cusack et al. 2021) while acknowledging the challenges of doing so. In order to fit to the 100-year time of GWP to soil C dynamics, as recommended by Petersen et al. (2013) and Smith et al. (2010), UKCP18 global projections based on a 60-km grid over the UK (horizon 2100) were used. The RCP 8.5 scenario represented a high emission scenario with stabilising CO_2_ emissions post-2100 (Meinshausen et al. 2011). However, RCP 2.6 was a low emission scenario, where CO_2_ emissions started declining by 2020 and fall to zero by 2100 (Meinshausen et al. 2011).

The RothC model tailored for managed grasslands under moist temperate conditions (Jebari et al. 2021) was used. The pedotransfer functions established by Weihermüller et al. (2013) were used to estimate all active C pools from initial measured SOC stocks. The initial inert organic matter pool was set to match the equation proposed by Falloon et al. (1998). The C inputs to the soil (which is mainly derived from plant) were derived from running the model to equilibrium. The estimation of above- and belowground residues was as follows. For partitioning the measured above-ground biomass, it was assumed that 65% was harvested or consumed by cows (Soussana and Lemaire 2014; and Poeplau 2016), and only 50% of the remaining fraction (i.e. of 17.5%) is turned over annually, becoming available for soil organic matter formation as aboveground residue (Schneider et al. 2006). Similarly, belowground residue was obtained by subtracting the aboveground residues from C inputs (estimated by running the model at equilibrium). To estimate rhizodeposition, a ratio of 0.5 between net rhizodeposition and belowground biomass typical for grassland species was adopted, as used in Pausch and Kuzyakov (2018). Finally, a carbon concentration of 45% of the plant biomass was assumed (Kätterer et al. 2012). For the decomposability of the monthly grass input, decomposable plant material (DPM), and resistant plant material (RPM) (the DPM:RPM ratio) for the different residue components (i.e. aboveground, belowground, and rhizodeposition), monthly measured neutral detergent fibre (NDF) values were used as a proxy for RPM and 100%–RPM% as a proxy for DPM (Jebari et al. 2021). The amount of cattle FYM was derived from measured data and converted to C, as suggested by Powlson et al. (2012).

### Potential mitigation scenarios

For the purposes of this study, a grouping of extant mitigation measures for beef systems in England was evaluated to identify the most promising management scenarios. In addition to the baseline scenario, the climate change impact of four alternative management strategies was evaluated. These comprised of the following:

- The application of a nitrification inhibitor during fertilisation (NI);
- The reduction of livestock density (LD) by 50%;
- The substitution of fertiliser nitrogen with symbiotically fixed nitrogen from legumes, in the form of white clover (WC), and;
- Anaerobic digestion (AD) of cattle manure.

In this sense, the selected interventions were the most efficient in terms of GHG mitigation according to existing scientific literature and can be modelled.

#### Anaerobic digestion

Anaerobic digestion of FYM is the process where organic material is broken down by microorganisms in the absence of oxygen. This process produces biogas, primarily composed of methane and carbon dioxide, which can be used as a renewable energy source. In this sense, the AD can help reduce GHG emissions from manure management by capturing methane that would otherwise be released into the atmosphere (Aguirre-Villegas et al. 2019). The remaining material, known as digestate, can be used as a nutrient-rich fertiliser (Nag et al. 2019). The estimated digestate was 1986 kg per year. The AD was applied to the housed FYM. We considered an anaerobic digester, with low leakage, high quality gastight storage, and best complete industrial technology, as defined in IPCC (2019) (Table S2). The methane conversion factor (MCF) was calculated according to IPCC (2019) recommendations. Emissions factors were altered for AD for both manure management and soil emissions (NAEI 2020) (Table S2). The C and N content of the digestate derived from AD was estimated using SIMSWASTE model equations at pre and post-anaerobic digestion stages (Pardo et al. 2017). Moreover, under this mitigation scenario, the plant growth was assumed as unaffected.

To capture the impacts of this system, digestate and biogas co-products were considered as follows: The digestate was assumed to be covered for sealed storage, and a gas collection system implemented to capture any methane produced for energy use elsewhere. Then, it was assumed to be applied to the grassland, forming part of the system, in replacement of the FYM. The biogas was assumed to be transferred in compressed form to avoid leakage and subsequently utilised on a more energy-intensive enterprise than the one within the system boundary which has no demand for gas, renewable or otherwise, as the barns are unheated. This hypothetical external enterprise (e.g. a pig breeding unit which requires notable amounts of energy to provide warmth for piglets post-weaning) is therefore responsible for the production and combustion of biogas in a suitable boiler. The *AGRIBALYSE* database recommendations were used to account for the AD, taking into account the impacts of infrastructure establishment and maintenance (Auberger et al. 2022; Avadi 2020). The latter were approximated from suitable proxies, such as adapting infrastructure data from similar technologies (e.g., storage) and rescaling *AGRIBALYSE* and *ecoinvent* infrastructure processes. Under this model design, it was assumed that an economic allocation of 76% biogas and 24% digestate since the co-products (i.e. digestate and biogas) have a different purpose and characteristic of interest, such as their energy content and their agronomic value.

#### Livestock density reduction

Moving towards extensification by reducing livestock densities has been underscored as a reliable mitigation measure in the UK (Sándor et al. 2018), although it is acknowledged that there may be unintended consequences for broader sustainability issues such as rural workforce and economies. These socioeconomic ramifications were beyond the scope of the present study. A reduction of livestock density by 50% (i.e. 0.52 instead of 1.04 LU ha^−1^) was assumed. Under this scenario, enteric fermentation emissions as well as manure emissions were assumed to be reduced by half, given their proportionality to the livestock density. However, soil emissions related to N_2_O and SOC changes were estimated, using IPCC (2019) guidelines and the RothC modified model, respectively (Jebari et al. 2021). N_2_O soil emissions and SOC changes depend on the manure input, as C inputs (Jebari et al. 2022). Under this scenario, C inputs derived from plant residues were assumed to remain consistent with the baseline since silage harvest is assumed to increase and be used by other farms.

#### Soil nutrient management

The application of the commonly used nitrification inhibitor (dicyandiamide (DCD)) was considered during fertilisation, as it is generally considered among the best available options to mitigate soil GHG emissions (Chadwick et al. 2018; Abalos et al. 2014). The recommended rate of application was 10 kg ha^−1^, as in Cardenas et al. (2019) and Chadwick et al. (2018). The emission factor for nitrate and DCD application to estimate direct N_2_O soil emissions (i.e. 0.54%) was used, as suggested by Cowan et al. (2020). However, the yield was considered the same, as proven by Hargreaves et al. (2021) and Cardenas et al. (2019) in trials in UK grassland systems. Under this management scenario, apart from the off-farm emissions considered under the baseline scenario (namely fertiliser production and transport energy), the emissions from the production and transport of DCD were also considered (see details in “*Nitrification inhibitor”* section of the Supplementary Information).

The introduction of symbiotically fixed nitrogen from legumes (e.g. white clover, *Trifolium repens*) was considered within the range of 30–50% sward coverage based on site-specific botanical surveys (Table S3). The introduction of such species into grasslands has been shown to be an effective mitigation measure (Fuchs et al. 2020). Under this scenario, measured data from an experiment carried out on the NWFP was considered. The scenario implies a change in plant properties. The FYM was applied to the field, as in the baseline. On-farm CH_4_ emissions (e.g. enteric fermentation and manure management) were modified according to the digestibility of the grass-clover mixture (72.8% compared with 71.7% under the baseline) (Table S1). Similarly, N_2_O emissions derived from manure management were adapted to the crude protein characteristics of the grass-clover mixture. N_2_O emissions derived from soil and SOC changes were changed according to the plant residue characteristics. However, under this management scenario, part of the off-farm emissions was omitted as there is no production or transport of fertilisers considered in this system.

### Uncertainty analysis

#### GHG emissions

One of the most limiting aspects of compiling an LCI is uncertainty associated with EFs or parameters linking nutrient inputs into the system with GHG outputs from the system (Pouliot et al. 2012). On real-world livestock farms, many factors can affect these ratios, including weather, soil, plant/animal genetics, management practice, and interactions between them. Despite this variability, the vast majority of carbon footprint studies adopt EFs derived outside the actual system boundary, most commonly in the form of parameters defined as part of IPCC guidelines. In this study, as mentioned previously, the majority of EFs were extracted from the UK inventory and local trials, e.g. the parameter for N_2_O emissions suggested by IPCC (2019), commonly known as EF1 (% fertiliser N lost as N_2_O), was extracted from Cowan et al. (2020). Quantitative estimates of the uncertainties in the emissions were calculated using a Monte Carlo simulation, as in McAuliffe et al. (2018) (See Table S4). This corresponds to the IPCC approach, discussed in the 2006 Guidelines (IPCC 2006), with no refinement in IPCC (2019). Regarding the emissions of input data related to the mitigation measures, namely AD and the application of a nitrification inhibitor, the pedigree approach subject to a qualitative assessment was adopted. Under this approach, SOC was assumed to be deterministic under the Monte Carlo simulation, and separately dealt with in the sensitivity analysis.

#### Soil organic carbon

Since C inputs are considered the main driver of SOC change (Wang et al. 2016) and in order to quantify the uncertainty in C inputs, a sensitivity analysis was run to estimate the SOC change (over 100 years) for the different soil treatments (i.e. permanent pasture as the baseline reference in this study and the grass and white clover mixture as a management scenario). For the sensitivity analysis, both the increase and the decrease in C inputs by ± 20% were considered (Smith et al. 2005; Dellar et al. 2018).

Climate change is known to affect plant growth and production through the interaction of different factors (i.e. temperature rise, precipitation change, and atmospheric CO_2_ enrichment) (Gamage et al. 2018) which, in turn, is influenced by management practices (Petersen et al. 2013). Given the negligeable effect of climate change on plant production in the Atlantic region of Europe, compared to other regions, and for simplicity, the effect of climate change on plant production and soil management was considered negligible. Several studies have assumed C input increases under climate change (e.g., Smith et al. 2005; Graux et al. 2012). However, this last assumption might be rather optimistic given rising evidence for negative effects of climate change on plant growth (Wiesmeier et al. 2016). Therefore, the possibility of stagnation, or even the reduction of C inputs, should be considered in SOC projections (Wiesmeier et al. 2016). According to Dellar et al. (2018), although climate change would affect the Atlantic zone of southwest England (with higher temperatures and CO_2_ concentrations and lower water availability), the impact on grassland productivity was rather negative, with an average decrease of 20%.

## Results and discussion

### Life cycle impact assessment (LCIA)

The LCA of beef production on permanent pasture resulted in an estimated global warming of 14.7 (SOC stock change projection under RCP 2.6) and 14.8 kg CO_2_-eq/kg LWG (SOC stock change projection under RCP 8.5). The estimated values were lower than those reported for the same site study by McAuliffe et al. (2018): 18.5 kg CO_2_-eq/kg LWG). This is explained by the fact that the calculations in our study were based on Tier 2 IPCC (2019) guideline refinements instead of IPCC (2019)guidelines used in the earlier study, combined with the inclusion of SOC changes, overlooked due to data limitations in the earlier study. Regardless, the climate change impact in this new study was greater than the corresponding average for Brazilian beef cattle production reported in Dick et al. (2021), which could be explained by the different systems and methodologies of both studies. Specifically, Dick et al. (2021) calculated the emissions and SOC stock changes using the Tier 2 IPCC (2019) method. The main hotspots of GHG emissions identified herein were CH_4_ arising from enteric fermentation (43%), followed by N_2_O emissions derived from soil (23%) and manure management (15.5%).

### SOC changes

The reduction in the rate of SOC change was 14.7% averaged among the different management scenarios comparing the RCP 2.6 projection with RCP 8.5 (Table S5). This reduction is due to the extreme climatic conditions of RCP 8.5, favouring higher SOC decomposition rates as reported in Jebari et al. (2023). Our study shows a role of C accumulation in the C footprint of a grazed beef production system in southwest England, leading to a mitigation efficacy of 3.8% under the baseline to 5% under the different management scenarios. Here, the mitigation potential is lower than the average reported in Jebari et al. (2022) for dairy production in Northern Spain under similar climatic conditions. This could be explained by the fact that in the latter study, a regional scale was considered for different municipalities presenting a variation in edaphoclimatic characteristics. The ongoing search for management strategies to increase the potential of soils to sequester C therefore continues to be relevant.

### Management scenarios

Our results suggested that based on the mitigation measures applied, improved manure management can offset the GHG emissions, leading to a more environmentally friendly livestock system. Indeed, the carbon footprint for 1 kg of beef LWG could be reduced by 7 to 26% for the beef system, by adopting the mitigation measures (namely nitrification inhibitor, white clover introduction, and AD). However, the livestock density reduction scenario showed the greatest climate change environmental impact compared with the remaining management scenarios (including the baseline). The carbon footprint per LWG under the livestock reduction scenario was increased by 24.5%, compared with the baseline.

#### Anaerobic digestion (AD)

**Fig. 2:**
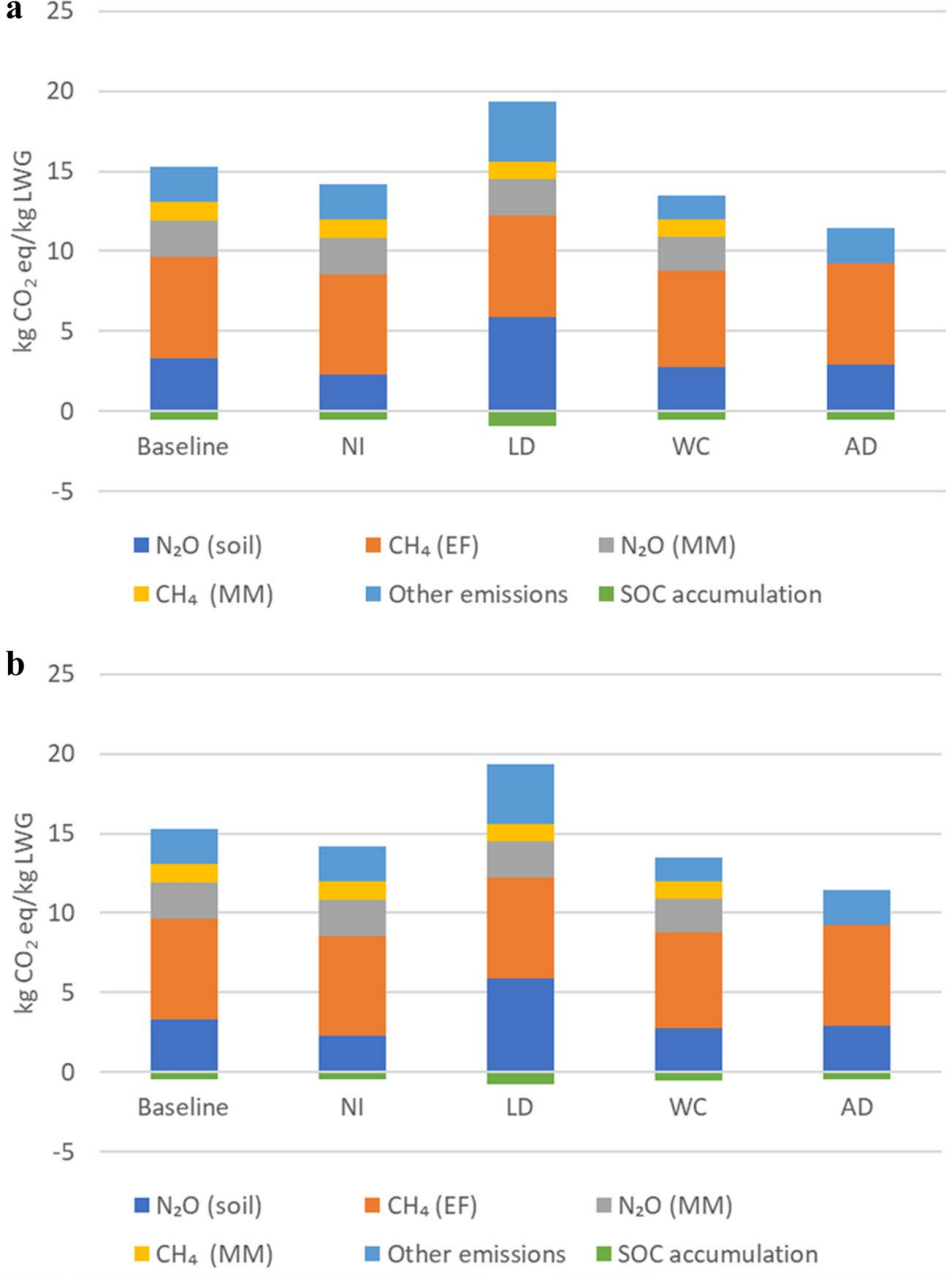
Breakdown of GHG emissions in kg CO_2_-eq/kg LWG under the baseline and different management scenarios. NI, nitrification inhibitor scenario; LD, livestock density reduction scenario (by 50%); WC, white clover introduction scenario; AD, anaerobic digestion scenario; EF, enteric fermentation; MM, manure management; other emissions include fertiliser production and transport (energy + seeds and pesticide for the WC scenario + Digestate and biogas for the AD scenario) for the beef production per liveweight gain ((a) SOC projected under RCP 2.6 climate change; (b) SOC projected under RCP 8.5 climate change)

The AD management scenario resulted in the lowest emissions derived from manure management, with a reduction of 99%, compared with the baseline scenario (Fig. 2). According to the results, the effect of digestate application instead of FYM was negligeable on SOC storage (Fig. 2). Moreover, soil N_2_O emissions derived from digestate application were reduced by 13% (Fig. 2). It is worth noting here that the yield was assumed to be unchanged as digestate fertilisers are supposed to not compromise grassland productivity (Walsh et al. 2018). Therefore, our study confirmed that AD can improve nutrient management (Bywater and Kush-Brandt 2022; Sanchez Rodriguez et al. 2018). Overall, the total climate change impact was reduced by 25%, under the AD scenario, compared with the baseline. The mitigation potential found in this study is in the range of the predictive modelling based on the IPCC refined methodology, which simulated up to 44% reduction of total commercial dairy farm emissions through the adoption of AD (Scott and Blanchard. 2021).

#### Livestock density reduction (LD)

Under the LD reduction management scenario, SOC intensity (per LWG) was increased by 63%, compared with the baseline, although the SOC change rate was reduced under the LD scenario, per ha. It is noteworthy here that the denominator of the carbon footprint, or the product output expressed in the functional unit (i.e. LWG), was halved. Therefore, the intensity of SOC accumulation increased, as the production decreased for the LD scenario. Livestock density was also identified as the main factor affecting net GHG emissions in the grasslands associated with livestock production under similar climatic conditions in northern Spain (Jebari et al. 2022). The reduction in livestock density was related to a reduction in manure application rates. However, the reduction in total LWG induced SOC increase per LWG. Similarly, under similar dietary conditions, methane emissions, derived from enteric fermentation, are primarily influenced by the livestock density (Liebig et al. 2010; Schönbach et al. 2012). Moreover, soil N_2_O losses can be mitigated effectively by reducing livestock density and/or N fertilisation rates (Sandor et al. 2018). However, the livestock-related emissions remained unchanged, compared with the baseline (Fig. 2), whereas soil N_2_O emissions intensity increased by 76%, compared with the baseline (Fig. 2). While the emissions related to the soil (e.g., lime production and application, fertilisers, energy, and transport) were maintained, the LWG production was halved. The effect of livestock density on animal intake and grass productivity was not studied since the same forage yield was assumed to be cut for silage purposes. Overall, the LD reduction scenario induced an increase of 25% in the carbon footprint for 1 kg of beef liveweight gain in the finishing operation, compared with the baseline.

#### Soil nutrient management: nitrification inhibitor (NI) and white clover introduction (WC)

With the adoption of the NI, soil N_2_O emissions were reduced by 33%. This estimate was close to Cowan et al. (2020), where DCD-treated ammonium nitrate reduced N_2_O emissions by approximately 28% on the basis of 20 years of experimental data in the UK and Republic of Ireland. The mitigation potential of the use of DCD on soil N_2_O emissions is also in the range established by Hargreaves et al. (2021) (i.e. 16% and 51%), depending on compaction depth and soil texture for the UK case. Indeed, the effectiveness of nitrification inhibitors (NIs) for reducing N_2_O emissions has been reported to vary considerably among different field studies in the UK because of different climate and soil conditions (Gilsanz et al. 2016; Cardenas et al. 2019). Together with NI application, it is important to ensure that the supply of N matches the need of the grassland system (Cardenas et al. 2019). Here, tools to mitigate emissions should be made available so farmers can apply the best strategy for applications at the right place and at the right time (Cardenas et al. 2019).

The white clover scenario showed a total offset of GHG emissions (compared with the baseline) of 12%. The introduction of white clover induced the highest SOC accumulation among the different management scenarios within the same total output production (i.e. LWG), with a slight increase of 2%, compared with the baseline (Fig. 2). The total yield of grass (kg dry matter per ha) decreased after the introduction of the white clover instead of fertilisation. However, the increase in SOC stocks in the grass-clover mixture, compared to the baseline fertilised permanent pasture, is explained by the increase in the belowground biomass. While N supply enhances aboveground biomass (Henry et al. 2005), in the case of nutrient shortage (or non-fertilisation), belowground biomass tends to be better developed in order to utilise available nutrients (Morgan et al. 2013). Moreover, in permanent grasslands, most carbon input is root-derived as the belowground residues are thought to contribute more carbon to organic matter than aboveground residues (Molina et al. 2001; Lorenz and Lal 2005; Poeplau 2016).

White clover, as a legume, improved not only the SOC content, but also the nitrogen use efficiency. Soil N_2_O emissions were reduced with white clover introduction by 18%, compared with the baseline. Symbiotically fixed nitrogen provides a supply of nitrogen for plants that is more synchronous to plant demand than occasional fertiliser applications (Carswell et al. 2019; Costa et al. 2021; Fuchs et al. 2020). However, a potential limitation of this mitigation measure can be the challenge of achieving high and persistent legume proportions, particularly in grasslands experiencing low sunlight or excessively cold growth periods (Barneze et al. 2022).

### Uncertainty analysis

#### Uncertainty analysis: Monte Carlo simulation

The results of the uncertainty analysis for the different GHG management scenarios are summarised in Table 2. The coefficients of variation (CV) of the C footprints (kg CO_2_ eq/kg LWG) for the different scenarios showed a low variability in most of the cases (less than 21% in 70% of the cases) and a moderate variability in 30% of the cases (less than ~ 32%) (Table 2). Therefore, the predictions of C footprints are reliable since the variation around the mean is not too wide.

**Table 2:** Monte Carlo simulation outputs of the C footprint (kg CO_2_ eq/kg LWG) under the different management scenarios at a 95% confidence interval

Management scenario	Mean	Median	SD	CV (%)
Baseline-RCP 2.6	15.06	15.00	3.10	20.55
Baseline-RCP 8.5	15.16	15.05	1.07	7.06
NI-RCP 2.6	13.96	13.89	2.15	15.37
NI-RCP 8.5	13.93	14.0	4.38	31.45
LD-RCP 2.6	18.88	18.74	2.44	12.94
LD-RCP 8.5	18.95	18.82	4.87	25.70
AD-RCP 2.6	11.28	11.17	2.73	24.20
AD-RCP 8.5	11.27	11.20	1.61	14.26
WC-RCP 2.6	13.29	13.14	1.91	14.37
WC-RCP 8.5	13.35	13.29	1.02	7.60

SD, standard deviation; CV, coefficient of variance; RCP 2.6 and RCP 8.5: representative concentration pathways: low emission scenario and high emission scenario, respectively; NI, nitrification inhibitor scenario; LD, livestock density reduction scenario (by 50%); WC, white clover introduction scenario; AD, anaerobic digestion scenario

#### Sensitivity analysis of SOC estimations

Under both grass swards (i.e., the permanent pasture and the grass-clover mix), the SOC stocks reduced/ or increased by ~ 7%, following the decrease/increase on C inputs, under both RCP 2.6 and RCP 8.5 projections. The decrease or increase, compared with the baseline, is in the range reported by Wiesmeier et al. (2016).

Under the permanent pasture, the 20% decrease or increase in C inputs showed a decrease or increase in the rate of SOC change of 19 and 21% under RCP 2.6 and RCP 8.5, respectively (Table 3). Under the grass and white clover mixture, the 20% decrease or increase in C inputs showed a decrease or increase in the rate of SOC change varying between 15 and 17% under RCP 2.6 and RCP 8.5, respectively (Table 3). Reporting impacts of management scenarios on changes in SOC when applying LCA, despite its minimal effect in this study, impart greater robustness for assessment of the sustainability of agri-food production systems.

**Table 3:** Changes in soil organic carbon stocks (Mg C ha^−1^) under two different grass swards (i.e., permanent pasture and grass-clover mix) on the NWFP

		RCP 2.6			RCP 8.5		
Grassland system	Measured or estimated SOC values	Plant C input decrease	No change	Plant C input increase	Plant C input decrease	No change	Plant C input increase
Permanent pasture (baseline)	Initial SOC stocks	53.78 ± 0.72	53.78 ± 0.72	53.78 ± 0.72	53.78 ± 0.72	53.78 ± 0.72	53.78 ± 0.72
	SOC stocks	65.91	71.27	76.63	63.39	68.47	73.56
	Annual SOC change rate	0.25	0.31	0.37	0.22	0.28	0.34
Mixture of grass and clover	Initial SOC stocks	38.88 ± 0.57	38.88 ± 0.57	38.88 ± 0.57	38.88 ± 0.57	38.88 ± 0.57	38.88 ± 0.57
	SOC stocks	64.22	68.98	73.74	61.77	66.30	70.82
	Annual SOC change rate	0.28	0.33	0.38	0.25	0.30	0.35

### Limitations

The originality of this research rests on assessing the partial life cycle of a grass-based beef cattle finishing system—‘cradle to finishing farm-gate’—in temperate climates, under different scenarios of GHG mitigation measures, using inventory analysis from the NWFP’s high-resolution records. However, the study inevitably involved limitations.

#### SOC changes

Regarding SOC dynamic estimation, uncertainty related to this work may be ascribed to changes in plant productivity and thus in C inputs under climate change scenarios (Dondini et al. 2018; Emadodin et al. 2021). The results for SOC changes following possible C input changes were assessed using a sensitivity analysis.

#### GHG emissions

In terms of GHG estimation, uncertainties could be induced by the IPCC Tier 2 method (Clark 2017). As the EFs are designed to be applicable to a wide spectrum of production environments within an agroecological zone, a considerable level of uncertainty surrounds each of these values. This, in turn, makes model-based estimates of on-farm GHG emissions less insightful than locally conducted field trials (Misselbrook et al. 2014), as the likelihood of detecting a statistically significant difference between treatments is lower when the descriptions of farming systems are less certain (Leinonen et al. 2012). However, following the Monte Carlo simulation, the results for the global warming were close to the mean values. Our findings could therefore be interpreted as a good indicator of the global warming of a grass-based beef cattle system in the study area.

Finally, regarding the input data related to the mitigation measures, namely AD and the application of a NI, the work herein referred to the scientific literature including our study site. However, more primary data on these mitigation measures based on local trials would be more reliable.

#### Future research

In order to analyse the contribution of animal sourced foods (and plant sourced foods) to a ‘cleaner’ planet more holistically, future studies need to explore major sustainability issues including biodiversity conservation and the protection of natural capital (achievable through the valorisation of local resources), maintenance of rural workforces and communities as well as animal and human welfare, while also exploring underrepresented complexities such as the unintended consequences of policy decision-making, including agri-environmental policy. Once these wider issues have been addressed using primary data, a driving force underpinning the ongoing NWFP (Segura et al. 2023 and McAuliffe et al. 2023), only then can the true value of food items be determined both at the product level and meal/diet level (e.g. multiple social, economic, and environmental indicators as well as their *potential* trade-offs) (Lee et al. 2021).

## Conclusion

The LCA approach enabled the assessment of interactions between different components of a highly instrumented beef production farm (in terms of feed production and quality, animal performance, manure management procedures, and material inputs and outputs, which are all measured/recorded meticulously and frequently) and its potential climate impacts under various scenarios. While beef production can contribute significantly to global warming, the different mitigation measures concerning manure management (anaerobic digestion) and grassland management (nitrification inhibitor, white clover introduction) were shown to be effective for reducing the global warming of the beef farming system. The findings, in this paper, emphasised the importance of reporting impacts of management scenarios on changes in SOC when applying LCA to assess the sustainability of agri-food production systems, despite their minimal effect in our specific case study.

## Supplementary Information

Below is the link to the electronic supplementary material.

Supplementary file1 (DOCX 29 KB)

## Data Availability

The data related to this paper will be available upon request.
