# Supplementary material for: Carbon footprints of greenhouse gas mitigation measures for a grass-based beef cattle finishing system in the UK
Source: Int J Life Cycle Assess. 2025 Jan 14;30(4):654–67. doi: 10.1007/s11367-025-02428-9 (PMC11923010; doi:10.1007/s11367-025-02428-9)
Supplement: Supplementary file 1 — Supplementary file1 (DOCX 29 KB) [file 11367_2025_2428_MOESM1_ESM.docx]

**Life Cycle Assessment of greenhouse gas** [**mitigation measures for**](https://www.sciencedirect.com/science/article/pii/S0377840111001660) **beef production grazing systems in temperate climates**

Asma Jebari^*^, Taro Takahashi, Michael R.F. Lee, Adrian L. Collins, Kevin Coleman, Alison Carswell, Carmen Segura, Laura Cardenas, and Graham A. McAuliffe

Asma Jebari: Net Zero and Resilient Farming, Rothamsted Research, North Wyke, Okehampton, Devon, EX20 2SB, United Kingdom

Taro Takahashi: Net Zero and Resilient Farming, Rothamsted Research, North Wyke, Okehampton, Devon, EX20 2SB, United Kingdom

Agri-Food and Biosciences Institute, Hillsborough, BT26 6DR, United Kingdom

University of Bristol, Langford, BS40 5DU, United Kingdom

Michael R.F. Lee: School of Sustainable Food and Farming, Harper Adams University, Edgmond, Shropshire, TF10 8NB, United Kingdom

Adrian L. Collins: Net Zero and Resilient Farming, Rothamsted Research, North Wyke, Okehampton, Devon, EX20 2SB, United Kingdom

Kevin Coleman: Net Zero and Resilient Farming, Rothamsted Research, Harpenden, AL5 2JQ, United Kingdom

Alison Carswell: Net Zero and Resilient Farming, Rothamsted Research, North Wyke, Okehampton, Devon, EX20 2SB, United Kingdom

Carmen Segura: Net Zero and Resilient Farming, Rothamsted Research, North Wyke, Okehampton, Devon, EX20 2SB, United Kingdom

Laura Cardenas: Net Zero and Resilient Farming, Rothamsted Research, North Wyke, Okehampton, Devon, EX20 2SB, United Kingdom

Graham A. McAuliffe: Net Zero and Resilient Farming, Rothamsted Research, North Wyke, Okehampton, Devon, EX20 2SB, United Kingdom

*Asma Jebari: [asma.jebari@rothamsted.ac.uk](mailto:asma.jebari@rothamsted.ac.uk); asmajebari543@yahoo.com

1. Baseline and potential mitigation scenarios

Table S1. Processes contribution (kg CO_2_-eq/kg of liveweight gain) for each scenario

| Name | Baseline | NI | LD | WC | AD |
| --- | --- | --- | --- | --- | --- |
| N₂O (soil) | 3.34 | 2.25 | 5.88 | 2.74 | 2.90 |
| CH₄ (EF) | 6.32 | 6.32 | 6.32 | 6.02 | 6.32 |
| N₂O (MM) | 2.27 | 2.27 | 2.27 | 2.17 |  |
| CH₄ (MM) | 1.14 | 1.14 | 1.14 | 1.03 |  |
| Ammonium nitrate | 1.07 | 1.07 | 2.14 | 0.00 | 1.07 |
| SOC accumulation (RCP 2.6) | -0.55 | -0.55 | -0.90 | -0.56 | -0.51 |
| SOC accumulation (RCP 8.5) | -0.49   \|  \| \| --- \| | -0.49 | -0.80 | -0.51 | -0.45 |
| Rapeseed | 0.20 | 0.20 | 0.20 | 0.39 | 0.20 |
| CO_₂_ (lime application) | 0.14 | 0.14 | 0.28 | 0.08 | 0.14 |
| Diesel combustion | 0.08 | 0.08 | 0.15 | 0.26 | 0.08 |
| Transport | 0.14 | 0.14 | 0.27 | 0.06 | 0.14 |
| Other fertilisers (P, K) | 0.08 | 0.08 | 0.16 | 0.10 | 0.08 |
| Lime production | 0.01 | 0.01 | 0.03 | 0.01 | 0.01 |
| Rye straw | 0.48 | 0.48 | 0.48 | 0.49 | 0.48 |
| Seeds and pesticide | |  |  | 0.11 |  |
| Digestate | |  |  |  | 0.03 |

NI: nitrification inhibitor scenario; LD: livestock density reduction scenario (by 50%); WC: white clover introduction scenario; AD: anaerobic digestion scenario; EF: enteric fermentation; MM: Manure management

1. Anaerobic digestion

Table S2. Manure management values for anaerobic digestion manure management scenario

| Parameter | Baseline | AD | Source |
| --- | --- | --- | --- |
| Ash | 0.08 | 0.08 | Equation 10.24 (IPCC 2019) |
| UE | 0.04 | 0.04 |  |
| Dietary GE | 18.45 | 18.45 |  |
| B_o_ | 0.18 | 0.18 |  |
| MCF(AD) | 17.31 | 1 | Updated from IPCC (2019) considering, Anaerobic Digester, Low leakage, High  quality gastight storage, best complete  industrial technology (Table 10.17) |
| MCF_pasture_ | 0.47 | 0.47 | Updated from IPCC (2019) considering cool temperate wet climate (Table 10.17) |
| C/N for cattle digestate |  | 6.54 | Hafner et al. (2022) |

1. Nitrification inhibitor

Nitrification Inhibitor Dicyandiamide (DCD): Since DCD is made from Cyanamide. The latter is produced by hydrolysis of [calcium cyanamide](https://eur01.safelinks.protection.outlook.com/?url=https%3A%2F%2Fen.wikipedia.org%2Fwiki%2FCalcium_cyanamide&data=05%7C01%7Casma.jebari%40rothamsted.ac.uk%7C7d73609bf23d41d2a19808db424a63e0%7Cb688362589414342b0e37b8cc8392f64%7C1%7C0%7C638176659504193012%7CUnknown%7CTWFpbGZsb3d8eyJWIjoiMC4wLjAwMDAiLCJQIjoiV2luMzIiLCJBTiI6Ik1haWwiLCJXVCI6Mn0%3D%7C3000%7C%7C%7C&sdata=GUjFvru0akPvg5SVY4giaKgKkxvNlv9E0UsHE6cncOk%3D&reserved=0), which in turn is prepared from [calcium carbide](https://eur01.safelinks.protection.outlook.com/?url=https%3A%2F%2Fen.wikipedia.org%2Fwiki%2FCalcium_carbide&data=05%7C01%7Casma.jebari%40rothamsted.ac.uk%7C7d73609bf23d41d2a19808db424a63e0%7Cb688362589414342b0e37b8cc8392f64%7C1%7C0%7C638176659504193012%7CUnknown%7CTWFpbGZsb3d8eyJWIjoiMC4wLjAwMDAiLCJQIjoiV2luMzIiLCJBTiI6Ik1haWwiLCJXVCI6Mn0%3D%7C3000%7C%7C%7C&sdata=%2B1UaF5ReoEy6XmTLy1P9SeXzm52Qr9GoaUfHkHbQeE8%3D&reserved=0) via the [Frank-Caro process](https://eur01.safelinks.protection.outlook.com/?url=https%3A%2F%2Fen.wikipedia.org%2Fwiki%2FFrank-Caro_process&data=05%7C01%7Casma.jebari%40rothamsted.ac.uk%7C7d73609bf23d41d2a19808db424a63e0%7Cb688362589414342b0e37b8cc8392f64%7C1%7C0%7C638176659504193012%7CUnknown%7CTWFpbGZsb3d8eyJWIjoiMC4wLjAwMDAiLCJQIjoiV2luMzIiLCJBTiI6Ik1haWwiLCJXVCI6Mn0%3D%7C3000%7C%7C%7C&sdata=OFWjeHBD2kUGjVvFLvK0bm%2BIGVgMy%2Bg1sAqVALsOxLw%3D&reserved=0)”. Therefore, given the non-availability of data on DCD, it was referred to Calcium carbide as a proxy for DCD *(Ecoinvent database)*.

The amount needed per kg of DCD, and thus Calcium carbide, was calculated by stoichiometry.

CaC_2_ + N_2_ = CaCN_2_ + C (Calcium carbide CaC_2_ reacts with nitrogen at high temperature to form calcium cyanamide CaCN_2_)

Since the recommendation is to use 10 kg/ha from DCD. For 21.6 ha, 216 kg are needed as DCD, equivalent by stoichiometry to 172.8 kg of Calcium carbide.

The transport needed for transporting 1kg of Calcium carbide, as defined in *Ecoinvent database* is as follows :

| Transport, freight train {RER}\| market group for transport, freight train \| Cut-off, U | 0.0438 | tkm |
| --- | --- | --- |
| Transport, freight, inland waterways, barge {RER}\| market for transport, freight, inland waterways, barge \| Cut-off, U | 0.0219 | tkm |
| Transport, freight, lorry, unspecified {RER}\| market for transport, freight, lorry, unspecified \| Cut-off, U | 0.1713 | tkm |

The emissions estimated from DCD, using SimaPro presented 0.038 kg CO_2_-eq/kg of liveweight gain.

1. White clover introduction

Table S3. Inventory for material inputs for the system with white clover introduction

| Input | Unit | WC |
| --- | --- | --- |
| Area | ha | 20.85 |
| Fertiliser area | ha | 20.52 |
| FYM area | ha | 17.97 |
| Yield | kg DM/ha | 10161 |
| Fertiliser |  |  |
| N | kg | 0 |
| P | kg | 400 |
| K | kg | 877 |
| Lime | kg | 2100 |
| Rapeseed expeller | kg | 3875 |
| Straw | kg | 39284 |
| Transport |  |  |
| Rapeseed (road) | tkm | 89 |
| Straw (road) | tkm | 2459 |
| Fertiliser (road) | tkm | 1445 |
| Pasture quality |  |  |
| DE | % | 76.62 |
| CP | % | 22.77 |
| Silage quality |  |  |
| DE | % | 68.94 |
| CP | % | 13.06 |

Where FYM is Farmyard manure, DE is digestibility and CP is Crude Protein

1. Uncertainty analysis

| Table S4. Distributions of uncertainty parameters assumed in Monte Carlo simulations, as in McAuliffe (2018) | | | | |
| --- | --- | --- | --- | --- |
| Emission source | Uncertainty | Distribution | Reference |  |
| *Animal/housing* |  |  | IPCC (2019) |  |
| Methane (EF and MM) | ± 20 % | Triangular |  |  |
| Nitrous oxide (direct MM) | SD^2^ = 2 | Lognormal |  |  |
| Nitrous oxide (indirect MM leaching) | -1500%/333% | Triangular |  |  |
| Nitrous oxide (indirect MM volatilisation) | SD^2^ = 5 | Lognormal |  |  |
| *Pasture* |  |  | IPCC (2019) |  |
| Nitrous oxide (direct) | SD^2^ = 3 | Lognormal |  |  |
| Nitrous oxide (indirect leaching) | -1500%/333% | Triangular |  |  |
| Nitrous oxide (indirect volatilisation) | SD^2^ = 5 | Lognormal |  |  |
| Carbon dioxide (lime) | -50%/0% | Triangular |  |  |

1. SOC changes

Table S5. Changes in soil organic carbon stocks (Mg C ha^-1^) for a combination of climate scenarios (RCP 2.6, RCP 8.5) and management scenarios (Baseline, NI, AD, LD, WC)

|  | Initial SOC stocks | Final SOC stocks | Annual SOC change rate |
| --- | --- | --- | --- |
| Baseline-RCP 2.6 | 53.8 | 71.3 | 0.20 |
| NI-RCP 2.6 | 53.8 | 71.3 | 0.20 |
| AD-RCP 2.6 | 53.8 | 69.0 | 0.18 |
| LD-RCP 2.6 | 53.8 | 66.3 | 0.14 |
| WC-RCP 2.6 | 40.3 | 69.0 | 0.33 |
| Baseline-RCP 8.5 | 53.8 | 68.5 | 0.17 |
| NI-RCP 8.5 | 53.8 | 68.5 | 0.17 |
| AD-RCP 8.5 | 53.8 | 66.3 | 0.14 |
| LD-RCP 8.5 | 53.8 | 63.7 | 0.11 |
| WC-RCP 8.5 | 40.3 | 66.3 | 0.30 |

NI: nitrification inhibitor scenario; LD: livestock density reduction scenario (by 50%); WC: white clover introduction scenario; AD: anaerobic digestion scenario
